# Role of Unfolded Protein Response in the Apoptosis Induced by Alphaarterivirus: IRE1α as an Essential Pathway for In Vitro Replication

**DOI:** 10.3390/v17101301

**Published:** 2025-09-25

**Authors:** Santiago Emanuel Colina, Macarena Marta Williman, María Soledad Serena, María Gabriela Echeverría, Germán Ernesto Metz

**Affiliations:** 1 Laboratorio de Virología, Centro de Microbiología Básica y Aplicada, Facultad de Ciencias Veterinarias, Universidad Nacional de La Plata, La Plata CP 1900, Buenos Aires, Argentina; scolina@fcv.unlp.edu.ar (S.E.C.); mwilliman@fcv.unlp.edu.ar (M.M.W.); solserena2000@fcv.unlp.edu.ar (M.S.S.); gecheverria@fcv.unlp.edu.ar (M.G.E.); 2 Consejo Nacional de Investigaciones Científicas y Técnicas (CONICET) CCT-La Plata, La Plata CP 1900, Buenos Aires, Argentina

**Keywords:** RNA virus, equine arteritis virus, ER stress, PERK, IRE1α, apoptosis

## Abstract

The perturbation of ER homeostasis by viral infection gives rise to the unfolded protein response (UPR), characterized by the activation of three signaling pathways. PERK, IRE1, and ATF6 have been identified as the primary mediators responsible for restoring homeostasis or leading to apoptosis in response to stress. *Alphaarterivirus equid*, known as equine arteritis virus (EAV), is a RNA virus with importance in the equine industry that could persist in semen and lead to abortions in pregnant mares. The present article explores the consequences of in vitro infection with the EAV Bucyrus strain on UPR. Employing RT-PCR, qPCR and Western blot, our investigation has revealed the activation of PERK and IRE1α pathways, whilst ATF6 has been suppressed. Furthermore, the p38α MAPK, caspase-12, and CHOP genes were found to be upregulated, demonstrating the induction of apoptosis. Finally, in the inhibition experiments, the PERK pathway was found to be implicated in the modulation of viral replication in the initial phases of infection. Conversely, the IRE1α pathway was identified as the predominant UPR pathway in EAV replication, as evidenced by the complete inhibition of replication observed in these experiments. Consequently, the further exploration of this UPR pathway is necessary to determine whether it can effectively suppress EAV replication.

## 1. Introduction

The equine arteritis virus (EAV), also known as *Alphaarterivirus equid*, is the causative agent of equine viral arteritis (EVA). EVA is a condition that affects members of the *Equidae* family worldwide [1]. Together with porcine respiratory and reproductive syndrome virus (PRRSV), they represent two important veterinary viruses in the *Arteriviridae* family. These viruses are included, together with *Coronaviridae*, in the *Nidovirales* order [2]. From both a clinical and economic perspective, the most significant consequences of EAV infection are abortion and viral persistence in the seminal fluid [3].

It is well established that positive RNA viruses, such as EAV, have the capacity to induce the formation of intracellular structures derived from different organelles. These structures, known as double membrane vesicles (DMVs), are crucial components of the virus’s replication process [4]. The endoplasmic reticulum (ER) is a crucial component in the replication and assembly of many RNA viruses. It is a critical organelle responsible for folding proteins that will become secreted or transmembrane, helping maintain protein homeostasis. Insufficiencies in the ER can lead to the accumulation of unfolded proteins leading to ER stress. Sensors in the ER detect folding imbalances and activate the unfolded protein response (UPR), a collection of pathways aimed at resolving stress. This response is critical for balancing the cellular fate between survival and death apoptosis [5].

In resting conditions, intraluminal chaperone glucose-regulated protein 78/immunoglobulin-binding protein (GRP78/BIP) is bound to three well-studied transmembrane ER proteins or sensors: PERK (Protein Kinase RNA-like ER Kinase), IRE1α (Inositol-Requiring Protein-1α), and ATF6 (Activating Transcription Factor 6) are the key players in this process. When ER stress occurs, BIP separates from the sensors, enabling the UPR to initiate [5,6].

The PERK pathway is initiated by the homodimerization and auto-phosphorylation of PERK, which subsequently leads to the phosphorylation of eIF2α. pEIF2α has been shown to induce the transcription of activation transcription factor 4 (ATF4), which plays a role in cell arrest by activating several genes implicated in stress response restoration, such as growth arrest and DNA damage-inducible 34 (GADD34), antioxidant response, and autophagy [6,7]. Additionally, ATF4 has been demonstrated to regulate the transcription of the transcriptional factor C/EBP homologous protein (CHOP), which can lead to apoptosis [8].

The activation of IRE1a leads to its dimerization and phosphorylation, which promote its nuclease activity and result in the non-canonical splicing of the X-box binding protein 1 mRNA (XBP1u), resulting in the production of the spliced form (XBP1s). XBP1 protein plays a crucial role in regulating ER-assisted degradation (ERAD), lipids, and chaperone synthesis as BIP, thereby contributing to the restoration of cell homeostasis [7]. Additionally, IRE1α interacts with TNF-receptor-associated factor 2 (TRAF2), which activates the apoptosis signal-regulating kinase 1 (ASK1). Consequently, this activates two mitogen-activated protein kinases (MAPKs): p38 and JNK. This activation leads to apoptosis through the activation of CHOP and several pro-apoptotic genes, such as Bax/Bak [8,9]. Furthermore, IRE1-TRAF2-ASK1 plays a crucial role in the activation of caspases, which leads to the proteolysis of inactive pro-caspase-12, resulting in the generation of active caspase-12. This, in turn, initiates a caspase cascade involving the caspase-9 and the effector caspase-3 [10,11].

The activation of the ATF6 branch is initiated by its translocation to the Golgi apparatus, where it is cleaved by proteases SP1 and SP2. This process generates the active form of ATF6, known as ATF6p50, which activates several pro-survival genes, including chaperones such as BIP, calnexin, and calreticulin, to enhance the folding capacity of the cell [6,12]. ATF6 pathway activation has been shown to induce the transcription of CHOP and XBP1 genes, indicating a cross-talk between UPR pathways [13,14].

Although several studies have investigated the modulation of the UPR following arterivirus infection particularly with PRRSV [15,16], there is a notable lack of research involving EAV. Nevertheless, ER stress has been shown to be induced upon EAV infection, as evidenced by the detection of caspase-12 in infected cell cultures [17].

Therefore, although indirect evidence supports the activation of the UPR following EAV infection, further studies are required to determine which specific UPR pathways are involved, how the shift toward apoptosis is regulated, and the extent to which UPR influences viral replication.

## 2. Materials and Methods

### 2.1. Cell Culture, Virus and Reagents

The African green monkey kidney cell line VERO CCL-81, used in all experiments, was obtained from the Asociación de Bancos Argentinos de Células (ABAC). Cells were cultured in Minimum Essential Medium (MEM) supplemented with 10% (*v*/*v*) fetal bovine serum (FBS), 0.04 mg/mL gentamicin, and 1.25 mg/mL amphotericin, and maintained at 37 °C in a humidified atmosphere containing 5% CO_2_. The cytopathic strain of equine arteritis virus, Bucyrus (hereafter referred to as EAV), was from our virus stock bank and used at a multiplicity of infection (MOI) of 1. Tunicamycin (Cell Signaling Technology, #12819, Danvers, MA, USA) was used as an inducer of endoplasmic reticulum (ER) stress at a final concentration of 7.5 μg/mL. Chemical inhibitors targeting the PERK and IRE1α pathways—GSK2606414 (#5165355MG) and 4-methylumbelliferone 8-carbaldehyde (4μ8c, #447910), respectively—were obtained from Thermo Fisher Scientific (Waltham, MA, USA). GSK2606414 was used at a final concentration of 200 nM, while 4μ8c was used at 50 μM. A polyclonal anti-phosphorylated IRE1α antibody (Thermo Fisher Scientific, #NB1002323SS) and a monoclonal anti-β-actin antibody (Cell Signaling Technology, #8H10D10) were used at a 1:1000 dilution. HRP-conjugated anti-rabbit and anti-mouse secondary antibodies were used at a dilution of 1:500.

### 2.2. Viral Stock and Infection Experiments

VERO cells were infected with an inoculum of EAV, and after one hour (h) of incubation at 37 °C for viral adsorption, cells were supplemented with a maintenance medium: MEM and 2% FBS. The infected cells were maintained at 37 °C until cytopathic effects (CPE) were observed in 70–80% of monolayers. Then, the supernatant was collected, aliquoted and frozen at −70 °C. Viral titration was conducted using tenfold serial dilutions for sextuplicate, and the titer, 10^6.75^, was obtained with the 50% tissue culture infectious doses (TCID50) assay using the Reed and Muench method.

For experimental infections, confluent VERO cell monolayers seeded in 12-well or 6-well plates were infected at a MOI of 1. After one hour of incubation at 37 °C, cells were supplemented with maintenance medium. Supernatants were collected at 8, 16, and 24 h post-infection (hpi). MOCK-infected cells and tunicamycin-treated cells (TUNI) were included as negative and positive controls, respectively.

### 2.3. RNA Extraction and Reverse Transcription Assay

Total cellular RNA was extracted using the High Pure RNA Isolation Kit from Roche (Indianapolis, IN, USA), following the manufacturer’s instructions. Then, 1 μg of total RNA was retro-transcribed using random hexamer from Biodynamic SRL (Ciudad de Buenos Aires, Argentina), RNasin Ribonuclease Inhibitor and M-MLV Reverse Transcriptase, both from Promega (Madison, WI, USA). The cDNA was stored at −20 °C until analysis.

### 2.4. qPCR Assay

For each condition, quantitative polymerase chain reaction (qPCR) was conducted in a final volume of 10 μL using iQ SYBR Green Supermix from Bio-Rad (Hércules, CA, USA), following the manufacturer’s instructions. All mRNA levels were obtained using the 2^−ΔΔCT^ method and the GAPDH gene was used as a housekeeping gene to normalize the values. The primers used in this study are listed in Table S1 [Supplementary Material]. The amplification of each specific product was analyzed by the melting curve obtained and 1.5% agarose gel electrophoresis.

### 2.5. PCR Assay

The activation of the IRE1α pathway was studied through the polymerase chain reaction (PCR) using primers targeting the different XBP1 transcripts described in Table S2 [Supplementary Material]. The PCR mix reaction was conducted in a final volume of 20 μL utilizing the Kit T-Plus Free ADN Polimerasa Resistente a Inhibidores 500 U from Inbio Highway SRL (Tandil, BA, Argentina). One microliter of each cDNA was used as template and the incubation conditions were as follows: 5 min at 95 °C, 35 cycles of 94 °C, 55 °C and 72 °C each for 30 s and a final extension step for 5 min at 72 °C. A 2% agarose gel was made for the visualization of the PCR product in ultraviolet light after staining with ethidium bromide. Band densitometry was performed using the Gel-Pro Analyzer software (Ver. 4.0). The qPCR GAPDH primers were used to normalize the bands.

### 2.6. Western Blotting Analysis

The UPR protein expression was evaluated through Western blot (WB) assay. Briefly, VERO cells growing in a 6-well plate were infected at MOI 1 with EAV for one hour, then the cells were supplemented with a maintenance medium and incubated at 37 °C with 5% of CO_2_ until harvesting at 8, 16 and 24 hpi. Negative MOCK and positive TUNI control cells were determined at 24 h. Following two washes with cold phosphate-buffered saline (PBS), the cells were treated with lysis buffer and boiled for 5 min. Protein was loaded and separated by sodium dodecyl sulfate polyacrylamide gel (SDS-PAGE) electrophoresis. The proteins were transferred on a polyvinylidene fluoride (PVDF) membrane and then blocked with PBS-Tween 20 1X + 5% of skim milk overnight at 4 °C. Next, the membranes were incubated with indicated primary antibody for 3 h at 37 °C, washed three times with PBS-Tween 20 1X for 5 min each and incubated with horseradish peroxidase (HRP)-conjugated secondary antibody for 3 h at 37 °C. After a final washing step with PBS, the bands were evidenced with incubation in PBS-3,3′-diaminobenzidine-H_2_O_2_ (30%) revelation solution.

### 2.7. UPR Inhibition and Viral Replication

The VERO cells were assayed in both the presence and absence of the inhibitor. We inhibited PERK and IRE1α pathways using GSK260414 and 4µ8c, respectively. Briefly, VERO was pre-incubated for one hour in the presence of each inhibitor, following the removal of the inhibitor cells, which were infected at a MOI of one with EAV for one hour subsequently, the viral inoculum was replaced with a maintenance medium in the presence of the inhibitor. The supernatant and cell portions were harvested at 8, 16 and 24 hpi. The progeny titer and the quantification of viral RNA was obtained as explained before.

### 2.8. Statistical Analysis

The data analysis was performed using the GraphPad Prism software (Ver. 8.0.2). The results are presented as the mean ± standard deviation (SD). The unpaired Student’s *t*-test was performed, with statistical significance evidenced as * *p* < 0.05, ** *p* < 0.01, *** *p* < 0.001 and **** *p* < 0.0001.

## 3. Results

### 3.1. ER Stress Response Following EAV Infection

**Figure 1:**
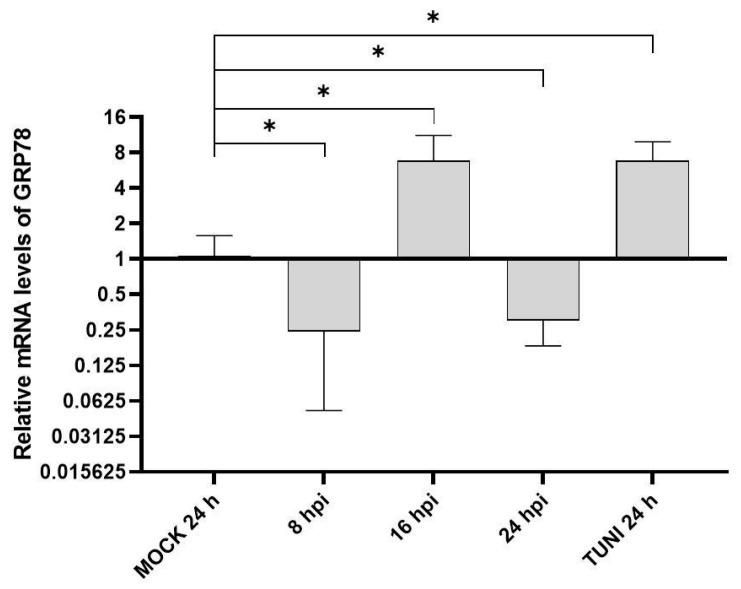
Activation of GRP78. The transcript levels of BIP were analyzed using RT-qPCR at 8, 16 and 24 hpi after infection of VERO cells with EAV at MOI 1. MOCK-infected and TUNI-induced cells were assayed at 24 h to serve as negative and positive controls, respectively. The mRNA values were relativized to the MOCK experiment and are presented as the mean ± SD after three independent experiments. * *p* < 0.05.

GRP78 is a well-known indicator of ER stress in cells. Therefore, we evaluated the transcription of this gene at different time points after infection with EAV (Figure 1).

An upregulation of GRP78 transcript levels was observed at 16 hpi, showing a four-fold increase relative to the MOCK control. Interestingly, transcript levels were downregulated at both early (8 hpi) and late (24 hpi) stages of infection.

### 3.2. UPR Modulation

**Figure 2:**
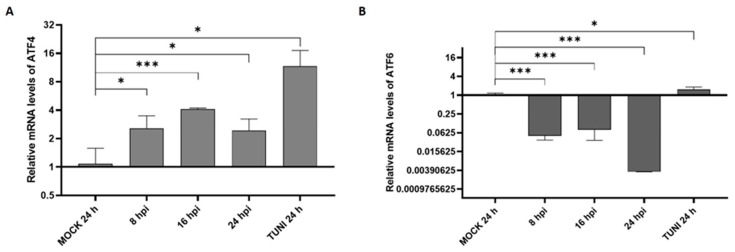
PERK and ATF6 pathway modulation. RT-qPCR was performed for the study of PERK through ATF4 (**A**) and ATF6 (**B**) at 8, 16 and 24 hpi. MOCK and TUNI treated cells were used as negative and positive controls, respectively. The bar values were obtained as explained before and represent the mean ± SD after three independent experiments. * *p* < 0.05, *** *p* < 0.001.

We first examined the potential modulation of the PERK and ATF6 branches, two principal pathways of the UPR, following EAV infection (Figure 2).

The results revealed the consistent upregulation of the PERK pathway across all time points analyzed. In contrast, ATF6 expression was downregulated, suggesting a possible suppression or shutdown of this UPR branch in response to infection.

**Figure 3:**
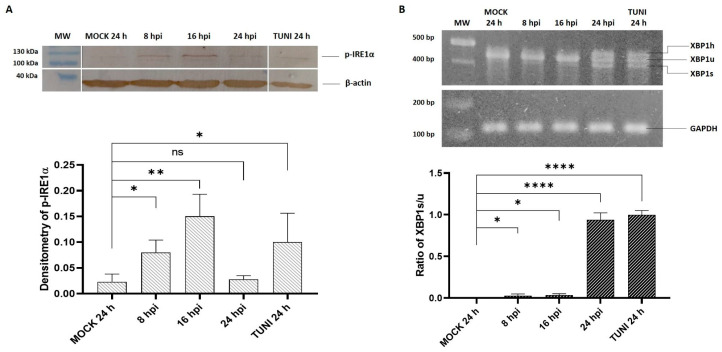
Activation of IRE1α pathway. (**A**) Western blot of IRE1α investigated with an antibody that recognizes the phosphorylation state of this sensor (p-IRE1α). (Up) A representative photograph of the PVDF membrane is shown, indicating the presence of the p-IRE1α and β-actin bands with sizes of ~115 and ~40 kilodalton, respectively. (Bottom) The densitometry of the pIRE1α bands was subsequently performed. The values were obtained by normalization with the β-actin band as a reference, with a value of 1. (**B**) The processing of XBP1 transcript was assayed through conventional RT-PCR. (Up) The representative photograph of 2% agarose gel with the different species indicated. (Botton) Densitometry of processed transcript, XBP1. The ratio of XBP1s/XBP1u using GAPDH as control with a value of 1. The bars represent the mean ± SD after three independent experiments. * *p* < 0.05, ** *p* < 0.01, **** *p* < 0.0001, ns = not significant.

The activation of the third UPR branch, the IRE1α pathway, was assessed for both evaluating phosphorylated IRE1α (p-IRE1α) levels via Western blot analysis and by quantifying the splicing of its downstream target transcript, XBP1, through RT-PCR (Figure 3).

The results demonstrate the activation of the IRE1α pathway at the multiple time points analyzed. Phosphorylation of the IRE1α sensor was prominently detected at 16 hpi, followed by a significant decline in phosphorylated IRE1α levels at 24 hpi (Figure 3A). Correspondingly, XBP1 processing showed a distinct activation at 24 hpi (Figure 3B). Notably, a third PCR band was observed at 24 hpi, corresponding to a hybrid form between the unspliced (XBP1u) and spliced (XBP1s) XBP1 transcripts, referred to as the XBP1 hybrid [18].

### 3.3. Apoptosis Indicators in EAV Infected Cells

**Figure 4:**
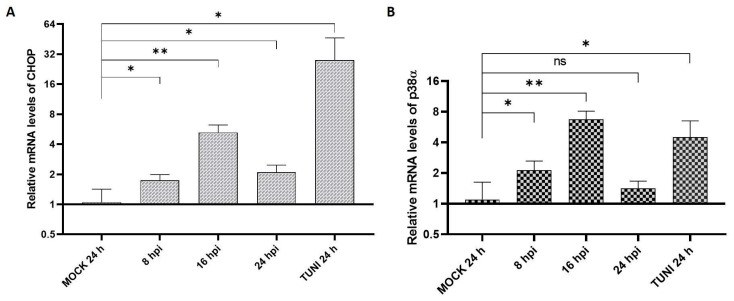
Transcription level of CHOP and p38α after EAV infection. The quantification of the mRNA levels for CHOP (**A**) and MAPK p38α (**B**) was studied at 8, 16, and 24 hpi by RT-qPCR. The MOCK experiment was used to relativize the values that are presented as the mean ± SD after three independent experiments. * *p* < 0.05, ** *p* < 0.01, ns = not significant.

CHOP and p38 MAPK are well-known transcriptional regulators involved in pro-apoptotic signaling and play critical roles in the balance between cell survival and cell death. Accordingly, we examined the mRNA expression levels of CHOP and p38α following EAV infection to assess their potential involvement in virus-induced apoptosis (Figure 4).

The results indicate the activation of the CHOP gene, with a peak induction of approximately six-fold at 16 hpi. Additionally, p38α MAPK expression showed an early upregulation at 8 and 16 hpi, reaching nearly an eight-fold increase compared to the MOCK control at 16 hpi. Taken together, these findings suggest a shift in the cellular response toward apoptosis in EAV-infected cells.

**Figure 5:**
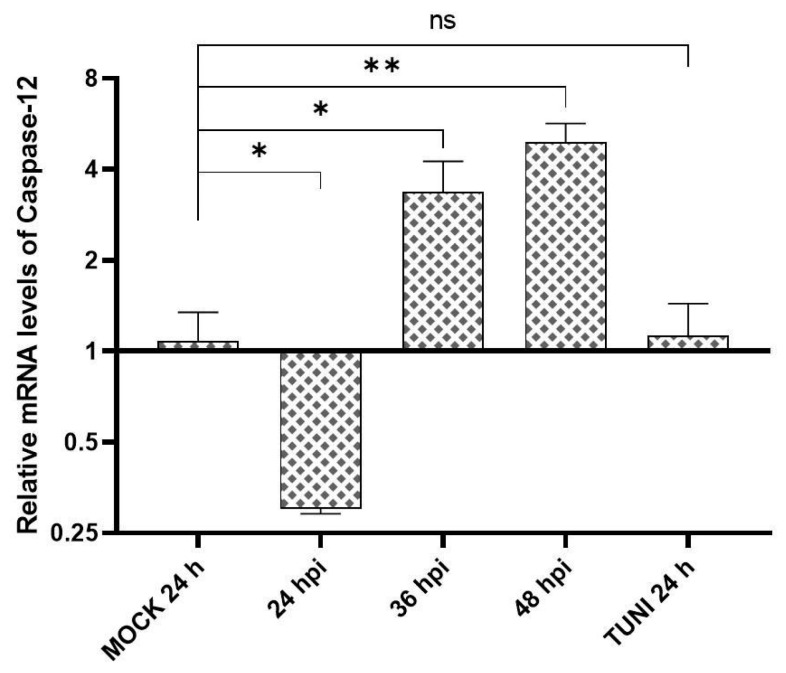
Caspase 12 activation. Levels of caspase-12 mRNA were investigated with RT-qPCR at 24-, 36- and 48-hpi. The MOCK and TUNI-treated cells were used as negative and positive controls, respectively. The values were relativized to MOCK well. The bars represent the mean ± SD after three independent experiments. * *p* < 0.05, ** *p* < 0.01, ns = not significant.

Caspase cascades are recognized as the central pathways mediating the activation of apoptosis. Among them, Caspase-12 plays a pivotal role in ER stress-induced apoptosis. Therefore, we assessed its activation at later time points during EAV infection (Figure 5).

Although caspase-12 expression was downregulated at early time points, a marked activation was observed at later stages post-infection.

### 3.4. EAV Replication and UPR Inhibition

**Figure 6:**
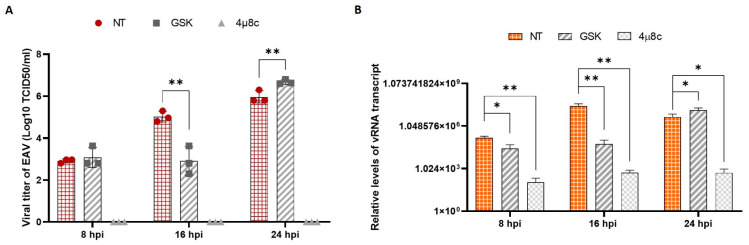
Inhibition of UPR pathways. The inhibitor effects on PERK or IRE1α pathways were assayed through viral titer and RT-qPCR in VERO infected cells in both conditions, not treated (NT) and in the presence of PERK (GSK) or IRE1α (4µ8c) inhibitors. (**A**) Viral progeny of EAV obtained with TCID50/mL assay after the infection of VERO cells in NT, GSK and 4µ8c conditions. (**B**) Quantification of viral RNA obtained in the same conditions with a focus on the viral N transcript. The values were normalized to the GAPDH gene and relativized to the MOCK experiment. The bars indicate the mean ± SD of three independent experiments. * *p* < 0.05, ** *p* < 0.01.

To investigate the relationship between EAV replication and the unfolded protein response (UPR), we selectively inhibited the PERK and IRE1α pathways and subsequently evaluated the impact on viral replication by measuring viral titers and viral RNA levels. Inhibition of the PERK pathway was achieved using GSK2606414 (GSK) at a final concentration of 200 nM, while the IRE1α pathway was inhibited with 4-methylumbelliferone 8-carbaldehyde (4µ8c) at a final concentration of 50 μM (Figure 6).

Our results indicate that the inhibition of the PERK pathway affects EAV replication at the initial stage of infection. Specifically, a reduction in viral RNA and progeny virus was observed at 16 hpi, suggesting a transient inhibition of viral replication. At 24 hpi, the inhibitory effect was not detectable. In contrast, the inhibition of the IRE1α pathway resulted in a severe and sustained reduction in viral RNA levels, as soon as 8 hpi, reducing 3–4 log when compared to the EAV-infected control. Consequently, no viral progeny was detectable at any time point analyzed.

## 4. Discussion

As intracellular parasites, viruses could induce endoplasmic reticulum stress. This is due to the ER’s need to produce viral proteins, which enables the rapid production of new progeny. The subsequent UPR activation after viral infection has been extensively studied for a number of viruses, especially RNA viruses [19,20]. Studies have shown that when ER stress occurs, the synthesis of chaperones is influenced by the activation of the UPR branches [5,7].

GRP78 is the primary chaperone regulator that dissociates from all UPR sensors to activate them as new molecules are synthesized to restore homeostasis [5]. Our investigation revealed an initially slight downregulation, followed by a clear peak activation at 16 hpi, comparable to that induced by the ER stress inducer tunicamycin (Figure 1). This GRP78 activation was also observed in PRRSV arterivirus [21,22]. Furthermore, a dose–response experiment revealed that higher levels of the minor envelope protein GP2a, from a virulent strain of PRRSV genotype 2, resulted in lower levels of GRP78, hence promoting viral RNA [23]. It would be worth investigating if this effect was also observed with EAV.

A global analysis of the PERK pathway through the ATF4 transcription factor demonstrated activation at early stages of infection followed by a decrease at 24 hpi (Figure 2A). This UPR pathway activation was also described in PRRSV and coronavirus infection [24,25]. However, it has been shown that ATF4 is retained in the cytoplasm by non-structural protein 2 of PRRSV, hindering the activation of target genes such as GADD34, which particularly negatively regulate the PERK activation system [23]. These studies also demonstrated that EAV and other RNA viruses do not retain ATF4 in the cytoplasm, making it feasible to hypothesize that there is negative feedback from PERK via GADD34, which would explain the decrease in PERK at 24 hpi.

In addition to PERK, our results also demonstrated the activation of the IRE1α pathway upon EAV infection (Figure 3). As previously described, activated IRE1α mediates the splicing of XBP1 mRNA via its endoribonuclease activity [7]. Employing Western blot analysis, we detected IRE1α phosphorylation, which peaked at 16 hpi (Figure 3A) and correlated with an increase in the spliced form of XBP1 mRNA observed at 24 hpi (Figure 3B). PRRSV infection experiments also evidenced the activation of IRE1α through both the phosphorylation of the sensor and XBP1 splicing process in both cell culture and PAM cells [15,21]. Furthermore, our study evidenced an XBP1 hybrid form (XBP1h) at 24 hpi and in TUNI-treated cells (Figure 3B), which is in concordance with other studies involving other RNA viruses [24,25].

Finally, the ATF6 branch is the only ER stress pathway that is not activated after EAV infection (Figure 2B). Remarkably, similar outcomes were observed following infection with PRRSV where the proteolytic cleavage of ATF6 does not occur, nor is there an increase in the expression of ATF6 target genes, such calnexin or calreticulin [21,23]. A particular case is the study by Catanzaro and Meng (2020), who used Western blot analysis and concluded that ATF6 was activated [26]. However, neither the full-length nor the cleaved form of ATF6 was detected, raising uncertainty regarding the evidence supporting ATF6 activation. All these findings suggest that this pathway could not be involved in the replication of arteriviruses.

It has been established that GRP78 can be activated by the three UPR pathways. The ATF6 pathway is widely recognized as a major regulatory pathway, with the ATF6p50 subunit being pivotal in the recognition of ER stress-responsive elements (ERSEs) in the promoters of various chaperones [27]. The IRE1α pathway, which has been demonstrated to mediate the synthesis of chaperones via XBP1, has also been identified as an important contributing factor [7].

Our study shows that EAV infection induces the complete inactivation of the ATF6 pathway and a delay in the activation of the IRE1α pathway. In addition, the PERK pathway has been identified as the only pathway that is activated, with peak activation at 16 hpi. On the basis of all these findings, we propose that the observation of GRP78 upregulation at 16 hpi could be due to the fact that only the PERK pathway has a stimulatory effect on the transcription of this chaperone. Moreover, evidence has demonstrated the involvement of gp2a PRRSV structural proteins in the degradation of GRP78 [23]. Consequently, it is plausible that this protein or another EAV structural protein could be associated with the reduction in GRP78 levels. Additional studies are needed to evaluate this proposal.

Under ER stress conditions, CHOP functions as a key pro-apoptotic mediator. Our study demonstrated that CHOP mRNA rises at all times (Figure 4A). Furthermore, we observed a p38α up-regulation (Figure 4B), evidencing the participation of MAPK pathways in the infection process of EAV. Previous works had demonstrated that both EAV and PRRSV induce another related pathway, NF-κB, as a way to induce cell death and promote its viral replication in response to cellular stress [27,28].

Apoptosis induced by EAV has been documented by several authors [29,30], with caspase-dependent cell death reported as a main consequence of infection [31]. Additionally, the involvement of caspase-12 in EAV infection was previously described [17]. In our study, we also detected caspase-12 expression at later time points (Figure 5). Although the detection was based on transcript levels, we hypothesize that both experimental results may be due to differences in the cell lines or MOI used.

Arterivirus infection triggers significant alterations in cell membranes, particularly within the endoplasmic reticulum, to facilitate the assembly of viral factories that are essential for replication [32]. In our study, we observed that EAV infection did not activate the ATF6 pathway, while clearly inducing the other two branches of UPR. Given the selective activation of PERK and IRE1α UPR pathways by EAV and PRRSV arteriviruses, we chose to inhibit each of them and analyze their role in viral replication (Figure 6).

The PERK pathway was inhibited using GSK, which demonstrated a significant delay in viral RNA replication and viral particle formation, particularly at 16 hpi. However, this reverses at 24 hpi, when there is a significant increase in viral RNA synthesis and viral particle formation, which is comparable to the uninhibited control. In contrast, the use of 4µ8c to inhibit the IRE1α pathway has been shown to result in a nearly complete suppression of viral RNA synthesis that influences the subsequent formation of new progeny.

According to Kublicka et al. (2025), at three hours post-infection, all EAV virions had fully uncoated within the host cells, thereby initiating the replication process [33]. Consequently, we inhibited IRE1α after 4 hpi, at the point when the replication/transcription process would be starting in order to determine the ability of 4µ8c to inhibit EAV replication at this time (Supplementary Figure S1).

Given that the levels of N mRNA expression in the EAV infected culture are close to 8 × 10^4^ copies after 8 hpi, the new inhibition of IRE1α at 4 hpi revealed 21 copies of N, as low as that previously obtained when IRE1α was inhibited at t = 0 (Supplementary Figure S1B). Conversely, we assessed the quantification of NSP1. The copy number of NSP1 in infected EAV cultures was 3.7 × 10^5^ at 8 hpi, while in the inhibited cultures the values at t = 0 and t = 4 were 12 and 16 copies, respectively (Supplemental Figure S1A).

Therefore, the results of the experiments demonstrate that, regardless of the time at which the IRE1α pathway is inhibited, the transcription of EAV NSP1 and N protein is never equal to that obtained in the replication cycle without inhibiting the IRE1α pathway. Hence, it is reasonable to infer that the genes regulated by IRE1α activation play an essential role in the EAV replication cycle. Research has shown that these genes are associated with the synthesis of chaperones and lipids. Therefore, we suggest that their inhibition could potentially counteract the formation of double-membrane vesicles, which are essential for providing a suitable environment for viral replication. It would be worthwhile to use gene knockdown analysis in future studies to assess the direct impact of XBP1 and other genes involved with lipid synthesis on viral replication.

The significance of this pathway in the replication of other members of the *Nidovirales* order, such as SARS-CoV-2, has also been demonstrated [34,35]. Consequently, based on the findings obtained, it can be concluded that arteriviruses trigger ER stress as a component of their pathogenic mechanism following cell infection. In particular, both EAV and PRRSV have been shown to preferentially activate the PERK and IRE1α pathways of the UPR, leading to ER stress-mediated apoptosis. The apoptotic process results in the release of different signals that recruit various immune cells, notably macrophages, which are known to be the primary target cells for arterivirus infection [36].

## 5. Conclusions

This study constitutes the first correlation between EAV infection and the development of UPR. Analysis of the UPR pathways revealed that the ATF6 branch is not activated following EAV infection, suggesting that it may not be required for viral replication. In contrast, our findings demonstrate that the IRE1α pathway plays a critical role in the EAV replication cycle. The inhibition of this pathway led to a complete reduction in both the viral RNA levels of N and NSP1 as well as progeny production, supporting its essential role in the infection process. Consequently, future research should focus on this signaling pathway and aim to identify viral proteins that interact with its components. Such studies may contribute to the development of novel therapeutic strategies against alphaarteriviruses. These insights pave the way for the development of novel therapeutic approaches against arteriviral infections, which hold significant relevance in veterinary medicine.

## Data Availability

Data are contained within the article and supplementary materials. Dataset available on request from the authors.

## Supplementary Materials

The following supporting information can be downloaded at: https://www.mdpi.com/article/10.3390/v17101301/s1. Table S1. List of primers used in RT-qPCR experiments. Table S2. List of primers used in RT-PCR experiments for the detection of both XBP1u/XBP1s form. Figure S1. 4µ8c influence in the replicative EAV cycle.

## References

[1] Balasuriya U.B., Go Y.Y., MacLachlan N.J. (2013). Equine arteritis virus. Vet. Microbiol..

[2] Cavanagh D. (1997). Nidovirales: A new order comprising Coronaviridae and Arteriviridae. Arch. Virol..

[3] Glaser A.L., Chirnside E.D., Horzinek M.C., de Vries A.A. (1997). Equine arteritis virus. Theriogenology.

[4] van der Hoeven B., Oudshoorn D., Koster A.J., Snijder E.J., Kikkert M., Bárcena M. (2016). Biogenesis and architecture of arterivirus replication organelles. Virus Res..

[5] He B. (2006). Viruses, endoplasmic reticulum stress, and interferon responses. Cell Death Differ..

[6] Hetz C., Zhang K., Kaufman R.J. (2020). Mechanisms, regulation and functions of the unfolded protein response. Nat. Rev. Mol. Cell Biol..

[7] Walter P., Ron D. (2011). The unfolded protein response: From stress pathway to homeostatic regulation. Science.

[8] Szegezdi E., Logue S.E., Gorman A.M., Samali A. (2006). Mediators of endoplasmic reticulum stress-induced apoptosis. EMBO Rep..

[9] Wang S., Binder P., Fang Q., Wang Z., Xiao W., Liu W., Wang X. (2018). Endoplasmic reticulum stress in the heart: Insights into mechanisms and drug targets. Br. J. Pharmacol..

[10] Szegezdi E., Fitzgerald U., Samali A. (2003). Caspase-12 and ER-stress-mediated apoptosis: The story so far. Ann. N. Y. Acad. Sci..

[11] Zhu H., Zhou H. (2021). Novel Insight into the Role of Endoplasmic Reticulum Stress in the Pathogenesis of Myocardial Ischemia-Reperfusion Injury. Oxid Med. Cell. Longev..

[12] Read A., Schröder M. (2021). The Unfolded Protein Response: An Overview. Biology.

[13] Hetz C., Papa F.R. (2018). The Unfolded Protein Response and Cell Fate Control. Mol. Cell.

[14] Shoulders M.D., Ryno L.M., Genereux J.C., Moresco J.J., Tu P.G., Wu C., Yates J.R., Su A.I., Kelly J.W., Wiseman R.L. (2013). Stress-independent activation of XBP1s and/or ATF6 reveals three functionally diverse ER proteostasis environments. Cell Rep..

[15] Huo Y., Fan L., Yin S., Dong Y., Guo X., Yang H., Hu H. (2013). Involvement of unfolded protein response, p53 and Akt in modulation of porcine reproductive and respiratory syndrome virus-mediated JNK activation. Virology.

[16] Chen W.Y., Schniztlein W.M., Calzada-Nova G., Zuckermann F.A. (2018). Genotype 2 Strains of Porcine Reproductive and Respiratory Syndrome Virus Dysregulate Alveolar Macrophage Cytokine Production via the Unfolded Protein Response. J. Virol..

[17] Metz G.E., Galindo I., Abeyá M.M., Echeverría M.G., Alonso C. (2016). Intrinsic, extrinsic and endoplasmic reticulum stress-induced apoptosis in RK13 cells infected with equine arteritis virus. Virus Res..

[18] Shang J., Lehrman M.A. (2004). Discordance of UPR signaling by ATF6 and Ire1p-XBP1 with levels of target transcripts. Biochem. Biophys. Res. Commun..

[19] Mehrbod P., Ande S.R., Alizadeh J., Rahimizadeh S., Shariati A., Malek H., Hashemi M., Glover K.K.M., Sher A.A., Coombs K.M. (2019). The roles of apoptosis, autophagy and unfolded protein response in arbovirus, influenza virus, and HIV infections. Virulence.

[20] Jheng J.R., Ho J.Y., Horng J.T. (2014). ER stress, autophagy, and RNA viruses. Front. Microbiol..

[21] Chen Q., Men Y., Wang D., Xu D., Liu S., Xiao S., Fang L. (2020). Porcine reproductive and respiratory syndrome virus infection induces endoplasmic reticulum stress, facilitates virus replication, and contributes to autophagy and apoptosis. Sci. Rep..

[22] Diao F., Jiang C., Sun Y., Gao Y., Bai J., Nauwynck H., Wang X., Yang Y., Jiang P., Liu X. (2023). Porcine reproductive and respiratory syndrome virus infection triggers autophagy via ER stress-induced calcium signaling to facilitate virus replication. PLoS Pathog..

[23] Gao P., Chai Y., Song J., Liu T., Chen P., Zhou L., Ge X., Guo X., Han J., Yang H. (2019). Reprogramming the unfolded protein response for replication by porcine reproductive and respiratory syndrome virus. PLoS Pathog..

[24] Echavarría-Consuegra L., Cook G.M., Busnadiego I., Lefèvre C., Keep S., Brown K., Doyle N., Dowgier G., Franaszek K., Moore N.A. (2021). Manipulation of the unfolded protein response: A pharmacological strategy against coronavirus infection. PLoS Pathog..

[25] Colina S.E., Williman M.M., Tizzano M.A., Serena M.S., Echeverría M.G., Metz G.E. (2024). Morbillivirus Canis Infection Induces Activation of Three Branches of Unfolded Protein Response, MAPK and Apoptosis. Viruses.

[26] Catanzaro N., Meng X.J. (2020). Induction of the unfolded protein response (UPR) suppresses porcine reproductive and respiratory syndrome virus (PRRSV) replication. Virus Res..

[27] Lee S.M., Kleiboeker S.B. (2005). Porcine arterivirus activates the NF-kappaB pathway through IkappaB degradation. Virology.

[28] Mottahedin A., Paidikondala M., Cholleti H., Baule C. (2013). NF-κB activation by equine arteritis virus is MyD88 dependent and promotes viral replication. Arch. Virol..

[29] Archambault D., St-Laurent G. (2000). Induction of apoptosis by equine arteritis virus infection. Virus Genes.

[30] Cholleti H., Paidikondala M., Munir M., Hakhverdyan M., Baule C. (2013). Equine arteritis virus induced cell death is associated with activation of the intrinsic apoptotic signalling pathway. Virus Res..

[31] Abeyá M.M., Metz G.E., Franco Cruz R., Correas I., Osorio F.A., Echeverria M.G. (2018). Equine arteritis virus cytopathic effect: Caspase-dependent cell death as the major consequence observed. J. Microbiol. Exp..

[32] Knoops K., Bárcena M., Limpens R.W., Koster A.J., Mommaas A.M., Snijder E.J. (2012). Ultrastructural characterization of arterivirus replication structures: Reshaping the endoplasmic reticulum to accommodate viral RNA synthesis. J. Virol..

[33] Kublicka A., Lorek D., Mikołajczyk-Martinez A., Chodaczek G., Chwirot A., Bażanów B., Matczuk A.K. (2025). Imaging flow cytometry reveals the mechanism of equine arteritis virus entry and internalization. Sci. Rep..

[34] Fernández J.J., Marín A., Rosales R., Penrice-Randal R., Mlcochova P., Alvarez Y., Villalón-Letelier F., Yildiz S., Pérez E., Rathnasinghe R. (2024). The IRE1α-XBP1 arm of the unfolded protein response is a host factor activated in SARS-CoV-2 infection. Biochim. Biophys. Acta Mol. Basis Dis..

[35] Oda J.M., den Hartigh A.B., Jackson S.M., Tronco A.R., Fink S.L. (2023). The unfolded protein response components IRE1α and XBP1 promote human coronavirus infection. mBio.

[36] Snijder E.J., Kikkert M., Fang Y. (2013). Arterivirus molecular biology and pathogenesis. J. Gen. Virol..

