# Supplementary material for: Role of Unfolded Protein Response in the Apoptosis Induced by Alphaarterivirus: IRE1α as an Essential Pathway for In Vitro Replication
Source: Viruses. 2025 Sep 25;17(10):1301. doi: 10.3390/v17101301 (PMC12568121; doi:10.3390/v17101301)
Supplement: Supplementary file 1 [file viruses-17-01301-s001.zip › viruses-3755239-supplementary.pdf]

Supplementary Material

Table S1. List of primers used in RT-qPCR experiments.

Use	Primers	Sequences	Size (pb)	Reference
Housekeeping	GAPDH-Fw	5′-AGGTCGGAGTCAACGGATTT-3′	112	[1]
	GAPDH-Rv	5′-TAGTTGAGGTCAATGAAGGG-3′	112	
ER stress	BIP-Fw	5′-ACCGCTGAGGCTTATTGGG-3′	147	[1]
	BIP-Rv	5′-TGCCGTAGGCTCGTTGATG-3′	147	
PERK	ATF4-Fw	5′-CCAACAACAGCAAGGAGGAT-3′	143	[2]
	ATF4-Rv	5′-GTGTCATCCAACGTGGTCAG-3′	143	
ATF6	ATF6-Fw	5′-CGAATAGCCCAGTGAA-3′	180	[3]
	ATF6-Rv	5′-ATCTCGCCTCTAACCC-3′	180	
MAPK	p38α-Fw	5′-GCCCAAGCCCTTGCACAT-3′	156	[4]
	p38α-Rv	5′-TGGTGGCACAAAGCTGATGAC-3′	156	
Apoptosis	Casp12-Fw	5′-CCAGGAACGTTCTAGATGGCA-3′	113	This study
	Casp12-Rv	5′-TCCAGCGTTGTTCACCATGA-3′	113	
Apoptosis	CHOP-Fw	5′-ACCAAGGGAGAACCAGGAAACG-3′	201	[5]
	CHOP-Rv	5′-TCACCATTCGGTCAATCAGAGC-3′	201	
EAV Bucyrus	N-Fw	5′- GGGGATCCAGCTTGTCGATGGCGTC-3′	333	[6]
	N-Rv	5′- TTGAGCTCAATATCCACGTCTTACGG-3	333	

The Caspase-12 primers were designed using the Primer-BLAST tool of the *National Center of Biotechnology Information* database, based on the predicted *Chlorocebus sabaeus* caspase-12 mRNA sequence (accession number XM_038005267.1).

Table S2. List of primers used in RT-PCR experiments for the detection of both XBP1u/XBP1s form.

Use	Primers	Sequences	Size (pb)	Reference
IRE1α	XBP1-Fw	5′- CCTTGTAGTTGAGAACCAGG -3′	440/414	[1]
	XBP1-Rv	5′- GGGGCTTGGTATATATGTGG -3′	440/414	

References

1. Wang, Y.; Li, J.R.; Sun, M.X.; Ni, B.; Huan, C.; Huang, L.; Li, C.; Fan, H.J.; Ren, X.F.; Mao, X. Triggering unfolded protein response by 2-Deoxy-D-glucose inhibits porcine epidemic diarrhea virus propagation. Antiviral Res. ***2014***, 106, 33-41. doi:10.1016/j.antiviral.2014.03.007
2. Wolfson, J.J.; May, K.L.; Thorpe, C.M.; Jandhyala, D.M.; Paton, J.C.; Paton, A.W. Subtilase cytotoxin activates PERK, IRE1 and ATF6 endoplasmic reticulum stress-signalling pathways. Cell. Microbiol. ***2008***, 10, 1775-1786. doi:10.1111/j.1462-5822.2008.01164.x

3. Zhang, C.; Hu, J.; Wang, X.; Wang, Y.; Guo, M.; Zhang, X.; Wu, Y. Avian reovirus infection activate the cellular unfold protein response and induced apoptosis via ATF6-dependent mechanism. Virus Res. **2021**, 297, 198346. doi:10.1016/j.virusres.2021.198346
4. Cheng, Y.; Sun, F.; Wang, L.; Gao, M.; Xie, Y.; Sun, Y.; Liu, H.; Yuan, Y.; Yi, W.; Huang, Z.; Yan, H.; Peng, K.; Wu, Y.; Cao, Z. Virus-induced p38 MAPK activation facilitates viral infection. Theranostics. **2020**, 10, 12223-12240. doi:10.7150/thno.50992
5. Echavarría-Consuegra, L.; Cook, G.M.; Busnadiego, I.; Lefèvre, C.; Keep, S.; Brown, K.; Doyle, N.; Dowgier, G.; Franaszek, K.; Moore, N.A.; Siddell, S.G.; Bickerton, E.; Hale, B.G.; Firth, A.E.; Brierley, I.; Irigoyen, N. Manipulation of the unfolded protein response: A pharmacological strategy against coronavirus infection. PLoS Pathog. **2021**, 17, e1009644. doi:10.1371/journal.ppat.1009644
6. Stadejek, T.; Bj Rklund, H.; Bascu Ana, C.R.; Ciabatti, I M.; Scicluna, M.T.; Amaddeo, D.; McCollum, W.H.; Autorino, G.L.; Timoney, P.J.; Paton, D.J.; Klingeborn, B.; Bel K, S. Genetic diversity of equine arteritis virus. J Gen Virol. **1999**, 80, 691-699. doi:10.1099/0022-1317-80-3-691

Supplementary Figure S1

4μ8c influence in the replicative EAV cycle

In order to examine the impact of IRE1*α* pathway suppression on the EAV replication process, a time-addition experiment was conducted in which the inhibitor was added at different times point following the infection. Following 8 hpi, the cells were harvested, and reverse transcription–quantitative PCR (RT-qPCR) was performed using primers specific to NSP1 and N genes (Figure S1).

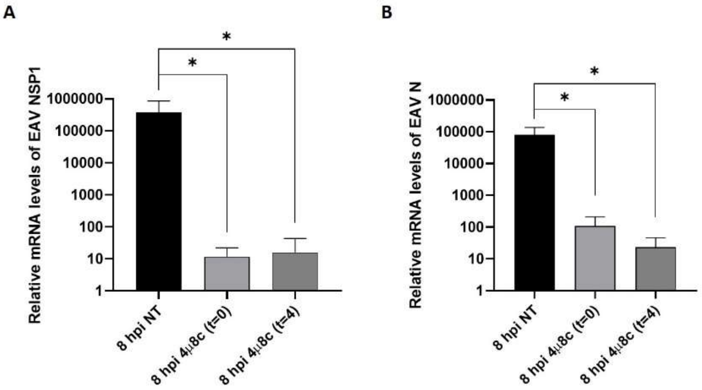

Figure S1. Quantification of specific viral EAV transcript after the inhibition of the IRE1*α* pathway. Vero cells were treated with 50 μM of IRE1*α* inhibitor, 4μ8c. The inhibitor was added prior to the infection experiment, at time equal zero (t=0) or 4 hours post-infection, at time equal 4 (t=4). GAPDH gene and the MOCK infected wells were used to normalized and relativized the values, respectively. **A.** Levels of EAV NSP1 transcript at different conditions. The primers were as follows: PFw: 5′-TATAAGCTTGCGACCATGGCAACCTTCTCCG-3′ and PRv: 5′-TATACTCGAGGCCGTAGTTGCCAGCAGG-3′. **B.** Levels of EAV N transcript at different conditions. The primers used are described in Table 1 of the Supplementary Materials. The bars represent the mean ± SD after three independent experiments and the statistical analysis was obtained after comparation with the not treated (NT) group. *p < 0,05.
